# Focal laser ablation as clinical treatment of prostate cancer: report from a Delphi consensus project

**DOI:** 10.1007/s00345-019-02636-7

**Published:** 2019-01-22

**Authors:** A. van Luijtelaar, B. M. Greenwood, H. U. Ahmed, A. B. Barqawi, E. Barret, J. G. R. Bomers, M. A. Brausi, P. L. Choyke, M. R. Cooperberg, S. Eggener, J. F. Feller, F. Frauscher, A. K. George, R. G. Hindley, S. F. M. Jenniskens, L. Klotz, G. Kovacs, U. Lindner, S. Loeb, D. J. Margolis, L. S. Marks, S. May, T. D. Mcclure, R. Montironi, S. G. Nour, A. Oto, T. J. Polascik, A. R. Rastinehad, T. M. De Reyke, J. S. Reijnen, J. J. M. C. H. de la Rosette, J. P. M. Sedelaar, D. S. Sperling, E. M. Walser, J. F. Ward, A. Villers, S. Ghai, J. J. Fütterer

**Affiliations:** 1 Department of Radiology and Nuclear Medicine, Radboud University Medical Center, Nijmegen, The Netherlands; 2 Desert Medical Imaging, Indian Wells, CA USA; 3 Department of Surgery, Imperial College London, London, UK; 4 Division of Urology, Department of Surgery, University of Colorado Denver School of Medicine, Aurora, CO USA; 5 L’Institut Mutualiste Montsouris, Paris Descartes University, Paris, France; 6 Department of Urology, AUSL Modena, Modena, Italy; 7 Molecular Imaging Program, National Cancer Institute, Bethesda, MD USA; 8 University of California San Francisco, San Francisco, CA USA; 9 Department of Urology, University of Chicago Medical Center, Chicago, IL USA; 10 Medizinische Universität Innsbruck, Innsbruck, Austria; 11 Urologic Oncology Branch, Center for Cancer Research, National Cancer Institute, National Institutes of Health, Bethesda, MD USA; 12 Department of Urology, Basingstoke Hospital, Hampshire Hospitals NHS Foundation Trust, Basingstoke, UK; 13 Sunnybrook Health Sciences Centre, Toronto, ON Canada; 14 Interdisciplinary Brachytherapy Unit, University of Lübeck, Lübeck, Germany; 15 Department of Urology, Kaplan Medical Center, Rehovot, Israel; 16 Department of Urology and Population Health, New York University and Manhattan Veterans Affairs Medical Center, New York, NY USA; 17 Department of Radiology, Ronald Reagan-UCLA Medical Center, Los Angeles, CA USA; 18 Department of Urology, University of California-Los Angeles, Los Angeles, CA USA; 19 Department of Urology, New York Presbyterian-Weill Cornell Medical College, New York, NY USA; 20 Section of Pathological Anatomy, Polytechnic University of the Marche Region, School of Medicine, United Hospitals, Ancona, Italy; 21 Department of Radiology and Imaging Sciences, Emory University School of Medicine, Atlanta, GA USA; 22 Department of Radiology, University of Chicago, Chicago, IL USA; 23 Department of Surgery, Duke University Medical Center, Durham, NC USA; 24 Department of Urology, Mount Sinai, New York, NY USA; 25 Department of Urology, Amsterdam UMC, Amsterdam, The Netherlands; 26 Department of Radiology, Sørlandet Hospital, Kristiansand, Norway; 27 Department of Urology, Istanbul Medipol University, Istanbul, Turkey; 28 Amsterdam UMC University Hospital, Amsterdam, The Netherlands; 29 Department of Urology, Radboud University Medical Center, Nijmegen, The Netherlands; 30 Sperling Prostate Center, Manhattan, NY USA; 31 Department of Radiology, University of Texas Medical Branch, Galveston, TX USA; 32 Division of Surgery, Department of Urology, University of Texas, Houston, TX USA; 33 Department of Urology, Lille University Medical Center, Lille, France; 34 University of Toronto, Toronto, ON Canada

**Keywords:** Laser focal therapy, Focal laser ablation, Prostate cancer, Consensus, Delphi

## Abstract

**Purpose:**

To define the role of focal laser ablation (FLA) as clinical treatment of prostate cancer (PCa) using the Delphi consensus method.

**Methods:**

A panel of international experts in the field of focal therapy (FT) in PCa conducted a collaborative consensus project using the Delphi method. Experts were invited to online questionnaires focusing on patient selection and treatment of PCa with FLA during four subsequent rounds. After each round, outcomes were displayed, and questionnaires were modified based on the comments provided by panelists. Results were finalized and discussed during face-to-face meetings.

**Results:**

Thirty-seven experts agreed to participate, and consensus was achieved on 39/43 topics. Clinically significant PCa (csPCa) was defined as any volume Grade Group 2 [Gleason score (GS) 3+4]. Focal therapy was specified as treatment of all csPCa and can be considered primary treatment as an alternative to radical treatment in carefully selected patients. In patients with intermediate-risk PCa (GS 3+4) as well as patients with MRI-visible and biopsy-confirmed local recurrence, FLA is optimal for targeted ablation of a specific magnetic resonance imaging (MRI)-visible focus. However, FLA should not be applied to candidates for active surveillance and close follow-up is required. Suitability for FLA is based on tumor volume, location to vital structures, GS, MRI-visibility, and biopsy confirmation.

**Conclusion:**

Focal laser ablation is a promising technique for treatment of clinically localized PCa and should ideally be performed within approved clinical trials. So far, only few studies have reported on FLA and further validation with longer follow-up is mandatory before widespread clinical implementation is justified.

**Electronic supplementary material:**

The online version of this article (10.1007/s00345-019-02636-7) contains supplementary material, which is available to authorized users.

## Introduction

The incidence of prostate cancer (PCa) is increasing with approximately 164,000 newly diagnosed cases in the US in 2018 [1]. Whole-gland therapy, i.e., radical prostatectomy (RP), external radiation therapy (RT), and brachytherapy are common treatment forms for PCa, providing excellent long-term efficacy but also come with treatment-related complications and side effects [2]. Studies reported urinary incontinence (13.4% and 4.4%, respectively), erectile function (EF) (75.7% and 71.9%, respectively) and bowel urgency (16.3% and 31.3%, respectively) within 5 years after RP or RT, resulting in a measurable decrease in quality of life [3]. Minimally invasive techniques are used for organ-confined PCa and are a novel strategy for targeted treatment, while preserving healthy tissue and subsequently reduce treatment-related morbidity [4]. Currently, a wide variety of energy sources seem to be capable for FT, i.e., focal laser ablation (FLA) cryosurgery, high intensity-focused ultrasound (HIFU), radiofrequency, microwave, and irreversible electroporation/nanoknife (IRE) [5–7].

Accurate imaging of PCa in conjunction with the associated anatomy is crucial for an effective and successful treatment. Multiparametric magnetic resonance imaging (mpMRI) is preferred for cancer localization and treatment planning over other imaging modalities due to its excellent soft-tissue contrast and multi-planar, anatomical imaging. It is also suitable for image guidance during treatment and long-term follow-up after FT [8]. During image-guided treatments, MRI-based temperature-mapping provides real-time feedback of the thermal distribution in the prostate and thereby increasing both precision and control. Also, MRI–transrectal ultrasound (TRUS) fusion appears to be safe and feasible for FLA guidance and monitoring [9].

The clinical use of FLA for localized PCa is not established yet and further evaluation is needed before it can be recognized as FT. The FLA thermal ablation technique is applied with a laser fiber and by raising the temperature > 60 °C, it results in direct focused cell death. Only few studies with small sample sizes and maximum follow-up of 1 year reported on the clinical use of FLA and, therefore, are lacking in long-term evidence [10–13]. However, they provide patient benefits supporting image-guided FLA as appealing technique and feasible and safe for minimal-invasive treatment, in men with low- and intermediate-risk PCa [Gleason score (GS) ≤ 3+4] who are eligible for both active surveillance (AS) and radical treatment. Targeted biopsies from the ablation zone showed no recurrence within 3 months after FLA in 96%. Systematic biopsies after 1 year showed a residual GS ≥ 3+3 in 11%. As a surrogate marker for efficacy, the mean prostate-specific antigen (PSA) has decreased by 40% following FLA [14, 15]. The aim of this international collaborative consensus project was to define the role of image-guided FLA as potential clinical treatment in patients with clinically localized PCa using the Delphi consensus method among experts in the field [16].

## Method

To achieve consensus among a panel of experts in the field, the Delphi method was used [16]. According to this method, online questionnaires were presented to participants during several rounds. The goal was to obtain consensus by reducing the range of answers after each round.

**Fig. 1:**
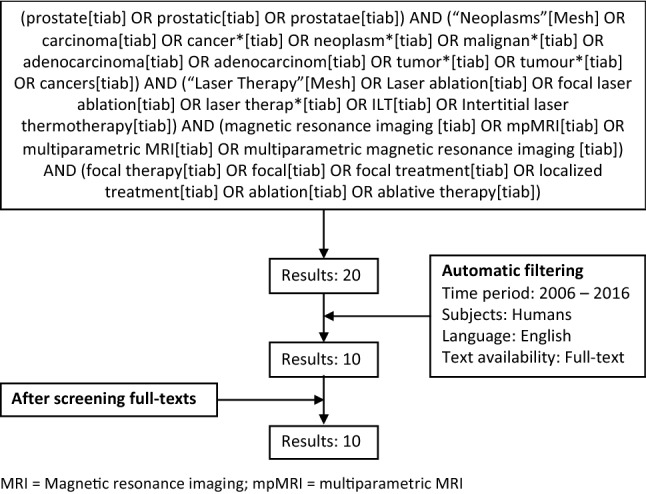
Overview of systematic research

A systematic literature search was conducted using the PubMed database on “prostate” (and synonyms), “cancer” (and synonyms), “focal laser ablation” (and synonyms), “magnetic resonance imaging” (and synonyms), and “focal therapy” (and synonyms), with filters: Full text, to 2016/07/01, Humans, English. This search yielded 20 articles (Fig. 1), while only 10 articles were eligible for controversial topic selection.

Panelists were selected based on the literature search and peer recommendations. The survey was conducted and presented in four subsequent rounds between 28 November 2016 and 14 June 2017, using online questionnaire software (www.surveymonkey.com; San Mateo, USA). Questionnaires were modified after each round based on responses and feedback. A (statistical) summary of the previous rounds was provided allowing the experts to re-evaluate their opinion. The level of agreement to achieve consensus was set at 70% and descriptive statistics were used to determine the response rate of each topic. The selected topics from the literature search were demographics, patient characteristics, role of biopsy/imaging in FLA, tumor size, outcome, and genomics. Online Resource 1 displays an overview of the questionnaires.

During a face-to-face meeting at the American Urological Association Annual Meeting on 13 May 2017, the results of the first three rounds were presented and inconclusive topics were discussed. Remaining questions were reworded and presented in a fourth round. Final results of the project were discussed during a meeting at the Radiological Society of North America (RSNA) on 26 November 2017.

## Results

**Fig. 2:**
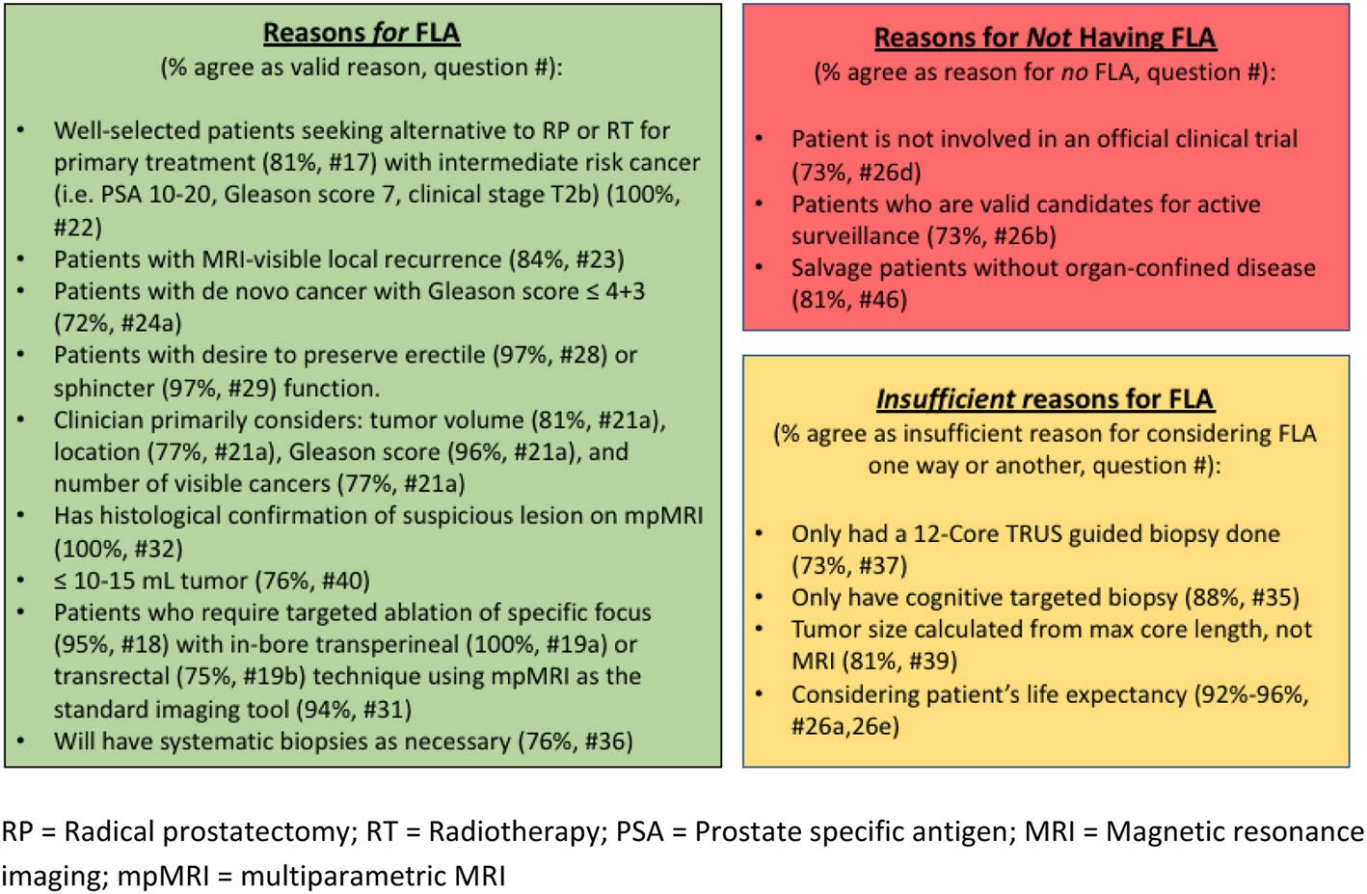
Summary of the results

Seventy-five international experts were invited, and 37 experts agreed to participate. Response rates were 100% (37/37), 70% (26/37), 68% (25/37), and 65% (24/37) for rounds 1–4, respectively. Fifty-one percent (19/37) were urologists, 38% (14/37) (interventional) radiologists, 3% (1/37) radiation oncologist, 3% (1/37) researcher, 3% (1/37) technical physician, and 3% (1/37) engineer. Seventy-eight percent (29/37) works in an academic hospital, 14% (5/37) in private practice, and 8% (3/37) in a major urban hospital. Eighty-six percent (32/37) reported PCa as their main field of expertise and 73% (27/37) treat more than 150 patients per year. One participant (3%) reported not to have treated any patients. Online Resource 2 contains the participants. Figure 2 shows the main results.

### Focal therapy

**Fig. 3:**
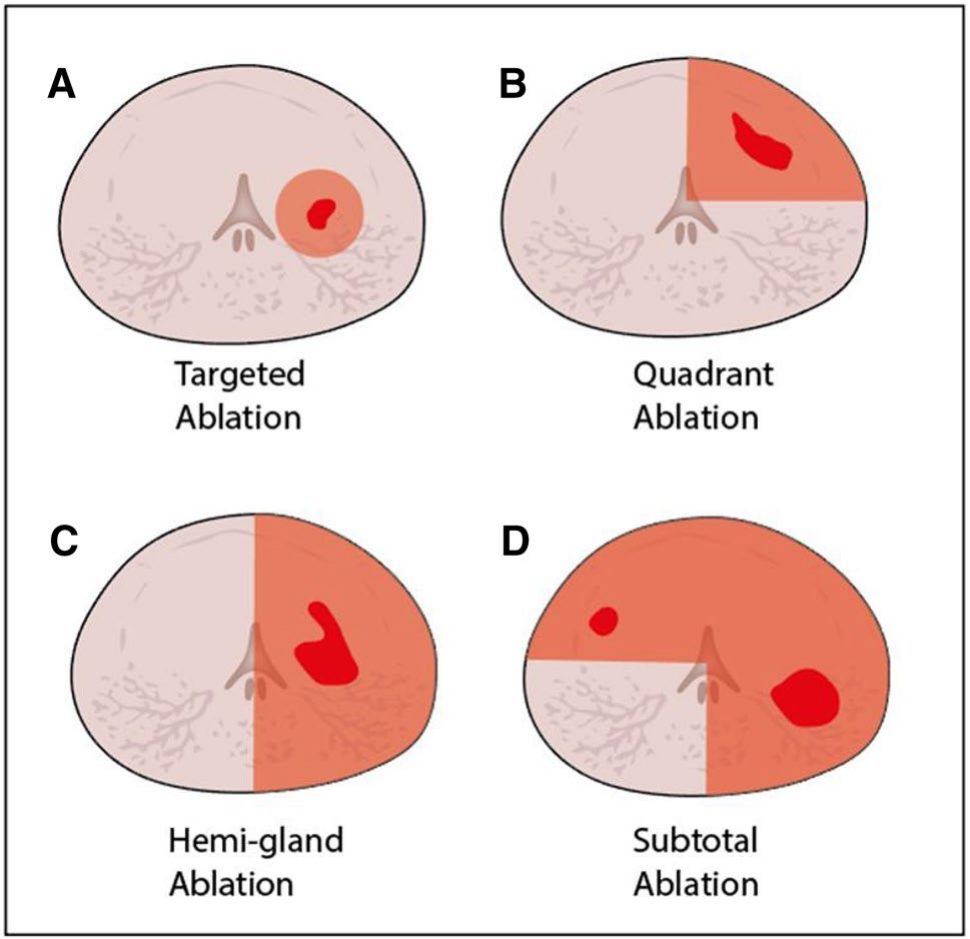
Axial schematic views of prostate gland with cancer foci (in red) and ablation zones (in orange); **a** targeted ablation, **b** quadrant ablation, **c** hemi-gland ablation, and **d** subtotal ablation

Clinically significant PCa (csPCa) is defined as any volume GS ≥ 3+4 (77%) and the lesion with both the largest volume and highest GS is considered the main index lesion (88%). Focal therapy for de novo PCa is defined as treatment of csPCa while leaving insignificant lesions under AS (88%). Subsequently, the same definition was used for FT as salvage treatment (80%). It is recommended to use FT as primary treatment of PCa in well-selected patients as alternative to RP or RT (81%). Focal laser ablation is optimal for targeting a specific focus (95%), rather than quadrant ablation (41%), hemi-gland ablation (24%) or subtotal ablation of the prostate (14%) (Fig. 3). Both, in-bore transperineal (100%) or in-bore transrectal (75%) approach are recommended for FLA. There was no consensus on the MRI–TRUS fusion approach. Preservation of EF (97%) and external urethral sphincter function (97%) are important reasons for choosing FLA over radical treatment. However, FLA is not appropriate for any patient outside of clinical trials (73%).

### Eligibility

Eligibility for FLA is determined by GS (95%), volume (81%), location (77%), and the number of MRI-visible and biopsy-confirmed cancers (77%). Lower urinary tract symptoms are not a contraindication for FLA (89%). The maximum prostate volume is not a primary determinant of eligibility for FLA (100%). There was no consensus on patient selection and recommended PSA cut-off value reasonable for FLA. Subsequently, panelists were of the opinion that PSA levels should not be considered or used for inclusion (54%). The maximum volume of cancer foci suitable for FLA should be based on MRI (81%), however, it is recommended not to exceed a tumor volume of 10–15 mL (76%). The panel recommended FLA in patients with low- and intermediate-risk (100%) or in patients with MRI-visible local recurrence (84%). It is also an acceptable strategy in patients with de novo PCa and GS ≤ 4+3 (72%). There was no consensus on the maximum GS in patients that require salvage therapy. However, salvage patients with only organ-confined disease are suitable candidates for FLA (81%). Patients who are AS candidate (GS 3+3) and otherwise do not require treatment are not eligible for FLA (73%). Life expectancy is not a primary determinant of suitability for FLA (92%). However, FLA should be offered when the life expectancy is less than 10 years, but only when treatment may delay local progression (96%). It should be applied to patients whose life expectancy, excluding the PCa diagnosis, is greater than their expected disease-specific mortality (88%).

### Biopsy and imaging

Histological confirmation is still required prior to FLA in the presence of a suspicious lesion (PI-RADS 4 or 5) on mpMRI (100%). Systematic biopsies remain necessary, even if an mpMRI suspicious lesion has already been sampled adequately by targeted biopsy (76%). However, 12-core biopsy alone is insufficient for patient selection for FLA (73%). The standard imaging tool for FT should be mpMRI (95%) and MRI–TRUS software fusion biopsy (86%) or in-bore MRI-guided biopsy (86%) are recommended to assess suspicious lesions. Cognitive-targeted biopsy was defined as not adequate to evaluate a lesion on mpMRI (88%).

### Outcome

According to the protocol, the prostate is regularly assessed by mpMRI and (systematic) biopsies after FLA. Treatment success is defined by a residual GS 3+3 obtained by random biopsy of the treatment area in combination with a negative mpMRI (95%). Residual in-field GS ≥ 3+4 on TRUS-guided biopsy is defined as treatment failure (84%), even with negative mpMRI (100%). Subsequently, a GS 4+3 based on in-field TRUS-guided biopsy a negative mpMRI is also considered as treatment failure (97%).

## Discussion

Consensus projects are valuable tools in fields where clinical evidence is still developing. The provided statements and recommendations can contribute to standardization of a therapy for clinical utilization. The results of our consensus project reflect the opinion of 37 experts with experience in the field of FT for localized PCa. Image-guided FLA can be offered to carefully selected patients as alternative to radical treatment. It is most favorable for targeting a specific focus in patients with GS ≤ 4+3 and MRI-visible, biopsy-proven PCa. Statements provided by this consensus project can be used as guidelines and recommendations for community-based (interventional) radiologists and urologists performing or referring patients for FLA.

Several other consensus projects have been conducted to provide the opinion of experts in the field. Postema et al. defined FT as targeting all identified tumors and with the aim to eradicate all csPCa (GS ≥ 3+4) [17]. Donaldson et al. described FT as focal ablation of the main index or dominant lesion and recommended FT for both targeted and quadrant ablations. They did not agree on the maximum tumor volume suitable for FT [18]. Our panel assigned similar definitions to both treatments of de novo PCa and salvage therapy. Commonly, the largest lesion is simultaneously the highest GS and consequently the main index lesion [19]. Our experts considered the lesion with both the largest volume and highest GS as the main index lesion and agreed on targeted ablation of a specific focus rather than quadrant ablation with a maximum tumor volume of 10–15 mL on mpMRI. Considering the MRI-invisible portion of a tumor is likely to extend significantly as the tumor volume increases, ablation of a tumor of this size requires significant margins. There has been a shift in eligibility criteria in recent years from low- to intermediate-risk due to growing confidence in AS for low-risk cancers [20–22].

Currently, a wide variety of energy sources are available for FT. This project focused on one technique, being image-guided FLA. Transrectal FLA is performed under local anesthesia and can, therefore, be offered as outpatient procedure. It only takes a few minutes to create a sharp ablation zone which results in relatively short procedure times and quick patient recovery [11]. Furthermore, multiple ablations can be performed during one session and repeated treatments, i.e., FLA or secondary whole-gland therapy are still viable options after FLA. Number of studies concluded FLA as feasible and safe while preserving urinary and sexual function [7, 12, 15]. Despite the promising results, efficacy and oncological outcomes are not well established. Image-guided FLA is limited to centers with experienced (interventional) radiologists or urologists and the use is still sporadic due to high costs and lack of insurance coverage. Moreover, the FLA procedure is less optimal for large-sized PCa compared to other modalities. Appropriate patient selection and assessing the efficacy of MRI-guided interventions remain a controversy. Other studies agreed on oncologic efficacy similar to our panel and stated that insignificant cancers in post-treatment biopsies do not need further treatment [18, 24]. Our experts agreed on in-bore MRI-guided biopsy being adequate for assessing a suspicious lesion on mpMRI or post-treatment biopsies similar to other studies [25, 26]. Cognitive-targeted biopsy was defined as not adequate. Remarkably, Yaxley et al. showed no significant difference between in-bore MRI-guided biopsy or cognitive-targeted biopsy and described the importance of identifying the lesion on mpMRI [23].

Number of uncertainties were identified and remain topics for further research. The role of FLA as salvage therapy was discussed during a face-to-face meeting [27]. Valerio et al. reported five series (*n* = 115) with follow-up ranging between 3 and 90 months, receiving FT (i.e., cryosurgery, HIFU, and MRI-guided brachytherapy) after RT failure and described the pad-free continence (87–100%), intact EF (29–40%), and the absence of csPCa in 92% [7]. Second, the use of both PSA and PSA-density (PSAD) as eligibility criteria for FLA remained undefined. Other studies considered a PSA ≤ 15 ng/mL eligible for FT and demonstrated PSAD as the most accurate predictor of PCa aggressiveness. The PSA was only used to predict seminal vesicle invasion [28, 29]. Third, there was no consensus on the MRI–TRUS fusion approach for FLA as experience has been limited to date. Natarajan et al. allowed accurate ablation of the lesion with MRI–TRUS fusion (*n* = 11). However, in-field biopsy after 6 months showed a GS 3+3 (*n* = 3) or csPCa (*n* = 4) [9].

Our study has several limitations. First, while using the Delphi method the results are based on the opinion of selected individuals and does not represent the PCa community at large. Expert selection can cause selection bias based on personal enthusiasm and preferences, since non-believers likely did not agree to participate. Also, all four questionnaires were not completed by every participating expert and the overall response rates were 65–100%.

## Conclusion

Focal laser ablation is a promising outpatient technique for treatment of clinically localized PCa in patients with GS ≤ 4+3 and MRI-visible, biopsy-proven cancer. This study has shown that despite the large number of low- and intermediate-risk disease, patient selection and eligibility criteria for FLA need to be evaluated based on which patients will benefit the most before clinical implementation is justified.

## Electronic supplementary material

Below is the link to the electronic supplementary material.

Questionnaire

List of panel experts
