# Supplementary material for: Focal laser ablation as clinical treatment of prostate cancer: report from a Delphi consensus project
Source: World J Urol. 2019 Jan 22;37(10):2147–53. doi: 10.1007/s00345-019-02636-7 (PMC6763411; doi:10.1007/s00345-019-02636-7)
Supplement: Supplementary file 1 — Questionnaire [file 345_2019_2636_MOESM1_ESM.docx]

**Electronic Supplementary Material 1** Questionnaire

Title: Focal laser ablation as clinical treatment of prostate cancer: report from a Delphi consensus project

Journal: World Journal of Urology

Authors: A van Luijtelaar, BM Greenwood, HU Ahmed, AB Barqawi, E Barret, JGR Bomers, MA Brausi, PL Choyke, MR Cooperberg, S Eggener, JF Feller, F Frauscher, AK George, RG Hindley, SFM Jenniskens, L Klotz, G Kovacs, U Lindner, S Loeb, DJ Margolis, LS Marks, S May, TD Mcclure, R Montironi, SG Nour, A Oto, TJ Polascik, AR Rastinehad, TM De Reyke, JS Reijnen, JJMCH de la Rosette, JPM Sedelaar, DS Sperling, EM Walser, JF Ward, A Villers, S Ghai, JJ Fütterer

Corresponding author:

A van Luijtelaar, [Annemarijke.vanluijtelaar@radboudumc.nl](mailto:Annemarijke.vanluijtelaar@radboudumc.nl), Radboudumc Nijmegen, P.O. Box 9101, 6500 HB Nijmegen, T: +31(0)24 361 4011, F: +31(0)24 354 0866

| Question | Response | |
| --- | --- | --- |
| Demographics | | |
| 3- Please provide your age (years):   - <30 - 30-40 - 40-50 - 50-60 - >60 | | **(round 1)**  0.0%  18.92%  37.84%  27.03%  16.22% |
| 4- What is your profession?   - Urologist - Oncologist - Radiotherapist - Radiologist - Interventional Radiologist - Prostate Cancer Researcher - Technical Physician - Interventional Urologist - Scientist/ Engineer | | **(round 1)**  48.65%  0.0%  2.70%  32.43%  5.41%  2.70%  2.70%  2.70%  2.70% |
| 5- In which kind of hospital do you work?   - Academic Hospital - Major urban Hospital - Minor urban Hospital - Private practice | | **(round 1)**  78.38%  8.11%  0.0%  13.51% |
| 6- Is Prostate Cancer your main field of expertise?   - No - Yes | | **(round 1)**  13.51%  86.49% |
| 7- How many prostate cancer related articles have you published in peer reviewed Journals?   - <10 prostate cancer related articles - 11-20 prostate cancer related articles - >20 prostate cancer related articles - N/A | | **(round 1)**  18.92%  16.22%  64.86%  0.0% |
| 8- How many patients are diagnosed with clinically localised prostate cancer per year in your center (no lymph-node involvement or metastasis)?   - <50 patients per year - 50-150 patients per year - 150-300 patients per year - >300 patients per year - N/A | | **(round 1)**  0.0%  27.03%  27.03%  45.95%  0.0% |
| 9- How many patients with prostate cancer per year do you treat (any treatment including active surveillance)?   - <10 patients per year - 10-20 patients per year - 20-50 patients per year - >50 patients per year - N/A | | **(round 1)**  2.70%  5.41%  16.22%  72.97%  2.70% |
| 10- How many of the treated patients have low-risk disease?   - <25% - 25-50% - 50-75% - >75% - N/A | | **(round 1)**  32.43%  40.54%  18.92%  5.41%  2.70% |
| 11- How many of the treated patients have intermediate-risk disease?   - <25% - 25-50% - 50-75% - 75-100% - N/A | | **(round 1)**  0.0%  59.46%  24.32%  13.51%  2.70% |
| 12- How many radical prostatectomies do you perform per year?   - None - <30 prostatectomies per year - 30-100 prostatectomies per year - >100 prostatectomies per year - N/A | | **(round 1)**  35.14%  5.41%  24.32%  21.62%  13.51% |
| 13- Consensus on a question is reached in case of:   - ≥ 50% agreement - ≥ 60% agreement - ≥ 70% agreement - ≥ 80% agreement | | **(round 2)**  3.85%  11.54%  **30.77%**  53.85% |
| Demographics | | |
| 14- How many patient ask you about focal therapy every month?   - None - <5 patients per month - 5-10 patients per month - 10-20 patients per month - >20 patients per month | | **(round 1)**  2.70%  29.73%  40.54%  8.11%  18.92% |
| 15- Is there any type of focal therapy available for your patients?   - No, at my knowledge - Only in my centre - In my region | | **(round 1)**  2.70%  48.65%  48.65% |
| 16a- Definition of significant PCa based on Gleason score?   - Gleason score ≥3+3 - Gleason score ≥3+4 - Gleason score ≥4+3 - Gleason score ≥4+4 - Other | | **(round 2)**  3.85%  **76.92%**  11.54%  0.0%  7.69% |
| 16b- Definition of the main index lesion?   - Largest lesion diameter (mm) - Largest lesion volume (mL) - Lesion with highest Gleason score - Both largest volume and highest Gleason score - Lesion with highest PI-RADS based on MRI - Other | | **(round 3)**  0.0%  0.0%  8.00%  **88.00%**  4.00%  0.0% |
| 16c- Which one amongst the following options better reflects/represents focal therapy for de novo prostate cancer?   - Treatment of the main index lesion whilst leaving untreated all other cancer foci, if any - Treatment of all significant lesions while leaving untreated and under surveillance all other lesions, if any - Treatment of all prostate cancer foci within the prostate gland whilst sparing the non-cancerous tissue - Focal therapy should not be considered as treatment for de novo prostate cancer | | **(round 2)**  3.85%  **88.46%**  7.69%  0.0% |
| 16d- Which one amongst the following options better reflects/represents focal therapy as salvage treatment?   - Treatment of the main index lesion whilst leaving untreated all other cancer foci, if any - Treatment of all significant lesions while leaving untreated and under surveillance all other lesions, if any - Treatment of all prostate cancer foci within the prostate gland whilst sparing the non-cancerous tissue - Focal therapy should not be considered as salvage treatment | | **(round 3)**  0.0%  **80.00%**  20.00%  0.0% |
| 17- Focal therapy should be considered as [multiple answers possible]:   - Salvage treatment (recurrence after surgery or radiation therapy) - Primary treatment in well-selected patients as an alternative to radical prostatectomy or radiation therapy - Primary treatment in well-selected patients as an alternative to active surveillance - It should be restricted to clinical trials populations at this time | | **(round 1)**  43.24%  **81.08%**  40.54%  45.95% |
| 18- Laser ablation is optimal for [multiple answers possible]:   - Targeted ablation of a specific focus - Quadrant ablation - Hemi-gland ablation - Subtotal ablation of the prostate | | **(round 1)**  **94.59%**  40.54%  24.32%  13.51% |
| 19a- In-bore transperineal approach is optimal for laser ablation:   - In-bore transperineal approach is not optimal - In-bore transperineal approach is optimal | | **(round 4)**  0.0%  **100.0%** |
| 19b- In-bore transrectal approach is optimal for laser ablation:   - In-bore transrectal approach is not optimal - In-bore transrectal approach is optimal | | **(round 4)**  25.00%  **75.00%** |
| 19c- MRI-TRUS fusion approach is optimal for laser ablation:   - MRI-TRUS fusion approach is not optimal - MRI-TRUS fusion approach is optimal | | **(round 4)**  66.67%  33.33% |
| Patient characteristics | | |
| 21a- Suitability of cancer foci for laser focal therapy should be based on [multiple answers possible]:   - PSA levels - PSA density - Tumor volume - Tumor location - Morphology - Gleason score - Patient characteristics - Life-expectancy - Number of MRI-visible, biopsy-confirmed cancers - All of the above | | **(round 2)**  50.00%  42.31%  **80.77%**  **76.92%**  30.77%  **96.15%**  53.85%  50.00%  **76.92%**  3.85% |
| 21b- In the presence of in-bore MRI biopsy or 12 core TRUS-guided biopsy proven clinically localized disease with negative bone scan and CT, laser focal therapy is reasonable for PSA:   - PSA <10 ng/mL - PSA <20 ng/mL - PSA is not used as an criteria for inclusion | | **(round 4)**  4.17%  41.67%  54.17% |
| 21c- In the presence of in-bore MRI biopsy or 12 core TRUS-guided biopsy proven clinically localized disease with negative PET scan and CT, with no clinical evidence of infection or inflammatory disease, PSA density (PSAD) should be considered for inclusion:   - PSAD should not be considered for inclusion - PSAD should be considered for inclusion | | **(round 4)**  50.00%  50.00% |
| 22- Laser focal therapy can be recommended for:   - Low-risk patients (PSA ≤10, Gleason score ≤ 6 or clinical stage T1-2a) - Intermediate-risk patients (PSA between 10-20, Gleason score 7 or clinical stage T2b) - High-risk patients (PSA >20, Gleason score ≥8 or clinical stage T2c-3a) | | **(round 2)**  0.0%  **100.0%**  0.0% |
| 23- Laser focal therapy is recommended in patients with local recurrence which is MRI-visible:   - Laser is not recommended in patients with local recurrence - Laser is recommended in patients with local recurrence - Laser is recommended in patients with MRI-visible local recurrence | | **(round 3)**  4.00%  12.00%  **84.00%** |
| 24a - Laser focal therapy is an acceptable strategy as treatment for de novo prostate cancer up to Gleason grade:   - Gleason score equal to 3+3 - Gleason score ≤3+4 - Gleason score ≤4+3 - Gleason score ≤4+4 - Gleason score >4+4 | | **(round 3)**  0.0%  12.00%  **72.00%**  12.00%  4.00% |
| 24b - Laser focal therapy is an acceptable strategy as salvage therapy up to Gleason grade:   - Gleason score equal to 3+3 - Gleason score ≤3+4 - Gleason score ≤4+3 - Gleason score ≤4+4 - Gleason score >4+4 | | **(round 3)**  0.0%  12.00%  8.00%  28.00%  52.00% |
| 25- The maximum prostate volume for laser focal therapy should be:   - ≤40 mL - >40 mL - Prostate volume should not be a primary determinant of eligibility for laser ablation | | **(round 3)**  0.0%  0.0%  **100.0%** |
| 26a- Laser focal therapy should be applied to patients with a life-expectancy of:   - ≤5 years - ≤10 years - ≤15 years - Life-expectancy should not be a primary determinant of eligibility for laser ablation | | **(round 3)**  0.0%  4.00%  4.00%  **92.00%** |
| 26b- Laser focal therapy should be applied to patients who are candidates for active surveillance:   - Focal therapy should not be applied to candidates for active surveillance - Focal therapy should be applied to candidates for active surveillance | | **(round 2)**  **73.08%**  26.92% |
| 26c- Laser focal therapy should be applied to patients whose life-expectancy excluding prostate cancer diagnosis is greater than their disease specific mortality:   - Focal therapy should not be applied to these patients - Focal therapy should be applied to these patients | | **(round 2)**  11.54%  **88.46%** |
| 26d- Laser focal therapy is an appropriate clinical treatment for any patient outside of clinical trials:   - Laser focal therapy is not an appropriate clinical treatment - Laser focal therapy is an appropriate clinical treatment | | **(round 2)**  **73.08%**  26.92% |
| 26e- Laser focal therapy should be offered to patients with a life-expectancy <10-years, where treatment may delay progression:   - Laser focal therapy should not be offered - Laser focal therapy should be offered | | **(round 3)**  4.00%  **96.00%** |
| 27- Lower urinary tract symptoms (LUTS) are a contraindication for laser focal therapy:   - LUTS are not a contraindication - LUTS are a contraindication | | **(round 1)**  **89.19%**  10.81% |
| 28- The potential for preservation of erectile function is an important reason for choosing laser focal therapy over radical treatment:   - Potential for preservation of erectile function is not an important reason - Potential for preservation of erectile function is an important reason | | **(round 1)**  2.70%  **97.30%** |
| 29- The potential for preservation of sphincter function is an important reason for choosing laser focal therapy over radical treatment:   - Potential for preservation of sphincter function is not an important reason - Potential for preservation of sphincter function is an important reason | | **(round 1)**  2.70%  **97.30%** |
| Role of biopsy/imaging in laser focal therapy | | |
| 31- mpMRI is a standard imaging tool for prostate focal therapy:   - mpMRI is not a standard imaging tool - mpMRI is a standard imaging tool | | **(round 1)**  5.41%  **94.59%** |
| 32- Histological confirmation is necessary prior to prostate laser focal therapy in the presence of a suspicious lesion (PI-RADS version 2 grade 4/5) on mpMRI:   - Histological confirmation is not necessary - Histological confirmation is necessary | | **(round 1)**  0.0%  **100.0%** |
| 33- MRI-TRUS software-fusion biopsy is adequate for assessing an mpMRI suspicious lesion:   - MRI-TRUS software-fusion biopsy is not adequate - MRI-TRUS software-fusion biopsy is adequate | | **(round 1)**  13.51%  **86.49%** |
| 34- In-bore MRI-guided biopsy is adequate for assessing a suspicious lesion on mpMRI:   - In-bore MRI-guided biopsy is not adequate - In-bore MRI-guided biopsy is adequate | | **(round 1)**  13.51%  **86.49%** |
| 35- Cognitive targeted biopsy is adequate for assessing a suspicious lesion on mpMRI:   - Cognitive targeted biopsy is not adequate - Cognitive targeted biopsy is adequate | | **(round 3)**  **88.00%**  12.00% |
| 36 - Systematic biopsies remaining necessary even if an MRI suspicious lesion has already been sampled adequately in a targeted manner:   - Systematic biopsies remain not necessary - Systematic biopsies remain necessary | | **(round 3)**  24.00%  **76.00%** |
| 37- A 12 core TRUS-guided biopsy alone is insufficient for patient selection for laser focal therapy:   - A 12 core TRUS-guided biopsy alone is not insufficient - A 12 core TRUS-guided biopsy alone is insufficient | | **(round 2)**  26.92%  **73.08%** |
| Tumor Size | | |
| 39- Tumor size should be based on:   - MR imaging - Maximum cancer core length | | **(round 1)**  **81.08%**  18.92% |
| 40- The maximum volume of cancer foci suitable for laser focal therapy on MRI imaging should be:   - <5 mL - 5-10 mL - 10-15 mL - >15 mL | | **(round 3)**  4.00%  8.00%  **76.00%**  12.00% |
| Outcome | | |
| 42- Tolerable untreated zone disease burden on TRUS-guided biopsy and negative mpMRI:   - Untreated Gleason Score 3+3 is not acceptable - Untreated Gleason Score 3+3 is acceptable | | **(round 1)**  5.41%  **94.59%** |
| 43- Tolerable untreated zone disease burden on TRUS-guided biopsy:   - Untreated Gleason Score 3+4 is not acceptable - Untreated Gleason Score 3+4 is acceptable | | **(round 1)**  **83.78%**  16.22% |
| 44- Tolerable untreated zone disease burden on negative mpMRI:   - Untreated Gleason Score 3+4 is not acceptable - Untreated Gleason Score 3+4 is acceptable | | **(round 2)**  **100.0%**  0.0% |
| 45- Tolerable untreated zone disease burden on TRUS-guided biopsy and negative mpMRI:   - Untreated Gleason Score 4+3 is not acceptable - Untreated Gleason Score 4+3 is acceptable | | **(round 1)**  **97.30%**  2.70% |
| 46- Salvage patients are suitable candidates for laser focal therapy for:   - Organ-confined disease only - Extraprostatic disease | | **(round 1)**  **81.08%**  18.92% |
| Genomics | | |
| 48- Tissue based genomic classifiers should be used for risk stratification:   - Tissue based genomic classifiers should not be used - Tissue based genomic classifiers should be used | | **(round 2)**  23.08%  **76.92%** |
| 49a- Known tissue based genomic classifiers (ERG, PTEN, SPINK1, etc.) should be used for research only:   - Known tissue based genomic classifiers are not for research only - Known tissue based genomic classifiers are for research only | | **(round 3)**  0.0%  **100.0%** |
| 49b- Unknown tissue based genomic classifiers should be used for research only:   - Unknown tissue based genomic classifiers are not for research only - Unknown tissue based genomic classifiers are for research only | | **(round 2)**  3.85%  **96.15%** |
