# Supplementary material for: Focal laser ablation as clinical treatment of prostate cancer: report from a Delphi consensus project
Source: World J Urol. 2019 Jan 22;37(10):2147–53. doi: 10.1007/s00345-019-02636-7 (PMC6763411; doi:10.1007/s00345-019-02636-7)
Supplement: Supplementary file 2 — List of panel experts [file 345_2019_2636_MOESM2_ESM.docx]

**Electronic Supplementary Material 2** List of panel experts

Title: Focal laser ablation as clinical treatment of prostate cancer: report from a Delphi consensus project

Journal: World Journal of Urology

Authors: A van Luijtelaar, BM Greenwood, HU Ahmed, AB Barqawi, E Barret, JGR Bomers, MA Brausi, PL Choyke, MR Cooperberg, S Eggener, JF Feller, F Frauscher, AK George, RG Hindley, SFM Jenniskens, L Klotz, G Kovacs, U Lindner, S Loeb, DJ Margolis, LS Marks, S May, TD Mcclure, R Montironi, SG Nour, A Oto, TJ Polascik, AR Rastinehad, TM De Reyke, JS Reijnen, JJMCH de la Rosette, JPM Sedelaar, DS Sperling, EM Walser, JF Ward, A Villers, S Ghai, JJ Fütterer

Corresponding author:

A van Luijtelaar, [Annemarijke.vanluijtelaar@radboudumc.nl](mailto:Annemarijke.vanluijtelaar@radboudumc.nl), Radboudumc Nijmegen, P.O. Box 9101, 6500 HB Nijmegen, T: +31(0)24 361 4011, F: +31(0)24 354 0866

| Name | Country |
| --- | --- |
| **Ahmed, HU** | United Kingdom |
| **Barqawi, A** | United States |
| **Barret, E** | France |
| **Bomers, JGR** | The Netherlands |
| **Brausi, MA** | Italy |
| **Choyke, PL** | United States |
| **Cooperberg, MR** | United States |
| **Eggener, S** | United States |
| **Feller, JF** | United States |
| **Frauscher, F** | Austria |
| **Fütterer, JJ** | The Netherlands |
| **George, AK** | United States |
| **Ghai, S** | Canada |
| **Greenwood, BM** | United States |
| **Hindley, RG** | United Kingdom |
| **Jenniskens, SFM** | The Netherlands |
| **Klotz, L** | Canada |
| **Kovacs, G** | Germany |
| **Lindner, U** | Israel |
| **Loeb, S** | United States |
| **Margolis, DJ** | United States |
| **Marks, LS** | United States |
| **Mcclure, T** | United States |
| **Montironi, R** | Italy |
| **Nour, SG** | United States |
| **Oto, A** | United States |
| **Polascik, TJ** | United States |
| **Rastinehad, AR** | United States |
| **De Reijke, TM** | The Netherlands |
| **Reijnen, JS** | Norway |
| **De la Rosette, JJ** | The Netherlands |
| **Sedelaar, JPM** | The Netherlands |
| **Sperling, DS** | United States |
| **Stuart, M** | United States |
| **Villers, A** | France |
| **Walser, EM** | United States |
| **Ward, JF** | United States |
